# Headplate Installation and Craniotomy for Awake In Vivo Electrophysiological Recordings or Two-Photon Imaging of the Mouse Inferior Colliculus

**DOI:** 10.21769/BioProtoc.4902

**Published:** 2023-12-20

**Authors:** Blom Kraakman, Sofja Solovjova, J. Gerard G. Borst, Aaron B. Wong

**Affiliations:** Department of Neuroscience, Erasmus MC, University Medical Center Rotterdam, NL-3015 GD Rotterdam, Netherlands

**Keywords:** Mouse, Inferior colliculus, Multielectrode recordings, Two-photon imaging, Long-term recording, Awake, Surgery

## Abstract

The inferior colliculus (IC) is an important processing center in the auditory system, which also receives non-auditory sensory input. The IC consists of several subnuclei whose functional role in (non-) auditory processing and plastic response properties are best approached by studying awake animals, preferably in a longitudinal fashion. The increasing use of mice in auditory research, the availability of genetic models, and the superficial location of the IC in the mouse have made it an attractive species for studying IC function. Here, we describe a protocol for exposing the mouse IC for up to a few weeks for in vivo imaging or electrophysiology in a stable manner. This method allows for a broader sampling of the IC while maintaining the brain surface in good quality and without reopening the craniotomy. Moreover, as it is adaptable for both electrophysiological recordings of the entire IC and imaging of the dorsal IC surface, it can be applied to answer a multitude of questions.

Key features

• A surgical protocol for long-term physiological recordings from the same or separate neuronal populations in the inferior colliculus.

• Optimized for awake in vivo experiments in the house mouse (*Mus musculus*).

## Background

The inferior colliculus (IC) is an auditory midbrain nucleus consisting of several sub-nuclei (Oliver, 2005). It receives both auditory and non-auditory input from several ascending and descending pathways (Lesicko et al., 2020). Research into the functional role of the IC in auditory processing would benefit from studies in awake animals. Moreover, responses of IC neurons have shown plasticity from stimulus exposure (Cruces-Solís et al., 2018) and activation of corticofugal projections (Suga, 2012). Longitudinal measurements would aid in the study of the underlying mechanism of these plastic changes. Although the mouse IC lies superficially, the thickness of the overlying interparietal bone and the height of the surrounding structures (cerebellum, cerebral cortex) make it difficult to establish stable access for long-term physiology. Here, we describe a protocol optimized for exposing the IC for stable, multi-day recordings by means of a chronic cranial window. This method can be used to either expose the dorsal surface for in vivo two-photon imaging (Wong and Borst, 2019) or make the entire IC readily accessible for repeated electrophysiological recordings (van den Berg et al., 2023).

The stability provided by this protocol allows a larger sampling of neurons within the same animal, repeated measurements of the same neurons over days (imaging), or a study of long-term changes in the timespan of days and weeks. This protocol was utilized to show that the cortical IC contains a tonotopic map, albeit different in organization than the central nucleus of the IC, characterized by a reversal of the characteristic frequency gradient in the rostromedial–caudolateral direction (Wong and Borst, 2019). Moreover, the same study confirmed non-auditory (in the form of spontaneous movement related) signal processing in the IC cortex. Another adaptation of the protocol showed that mice are able to distinguish amplitude modulations (AM) up to 512 Hz of an auditory stimulus. The set of IC neurons with the largest change in firing rate in response to this AM change are sufficient to explain the behavioral detection threshold (van den Berg et al., 2023).

The main advantage of this method is the possibility to gather data at multiple time points without having to reopen the craniotomy. This gives the opportunity to do multiple recordings in the same region over an extended period of time without implanting a recording probe or reopening the craniotomy, as well as recording at multiple sites to get a completer sample of the IC neuronal population. By sealing the craniotomy with a low viscosity silicone gel (Jackson and Muthuswamy, 2008), the risk of infection and amount of tissue regrowth are reduced. As a result, the brain surface is kept in better quality for measurements. A limitation is that a well is required to hold the silicone gel in place, which limits the angle of approach of a recording probe. The cranial window construct described here for chronic imaging provides the depth needed for mechanical stability while limiting the amount of glass in the light path. An inherent limitation of two-photon imaging is the limited depth of the imaging plane (Helmchen and Denk, 2005). Given the anatomical shape of the mouse IC, the imaging will be limited to the dorsocaudal part of dorsal and lateral cortices (Wong and Borst, 2019).

## Materials and reagents

**Reagents**

1. Superglue (e.g., UHU, catalog number: 62687)
2. Dental etching gel Etch Rite (38% phosphoric acid) (Pulpdent, catalog number: 185120)
3. Dental adhesive primers OptiBond FL 1 Prime (Kerr, catalog number: 25881E)
4. Dental adhesive OptiBond FL 2 Adhesive (Kerr, catalog number: 25882E)
5. Standard dental composite Charisma A1 (Heraeus, catalog number: 66056076)
6. Flowable dental composite Charisma Flow A1 (Heraeus, catalog number: 66015495)
7. Isoflurane
8. 70% ethanol
9. 0.9% NaCl (Baxter, catalog number: TKF7124)
10. Eye ointment (e.g., Alcon, Duratears Z, catalog number: 10308)
11. Silicone gel sealant (Dura-Gel, Cambridge NeuroTech, 2 components, catalog number: Dura-Gel or equivalent: DOWSIL^TM^ 3–4680 Silicone Gel Kit, DOW, catalog number: 3-4680)
12. Lidocaine spray, 100 mg/mL (e.g., Aspen, Xylocaine spray)
13. Buprenorphine (e.g., Dechra, Bupredine Multidose 0.3 mg/mL)
14. Carprofen (e.g., ASTfarma, Carporal 50 mg/mL)
15. 15% Mannitol (Sigma, catalog number: M4125-100g)

**Laboratory supplies**

1. Drill head (Ø 0.3 mm) (Komet Dental, catalog number: H71 104 003)
2. Fine disposable micro applicators (FHS Dental, catalog number:18-902T)
3. Sugi Sponge Points (Kettenbach GmbH & Co. KG, catalog number: 31603)
4. Cotton swabs
5. Haemostatic gelatin sponge (Spongostan Standard, Ferrosan Medical Devices, catalog number: MS0006)
6. Paper points (Henry Schein, catalog number: 9001217)
7. Clean round headplate (manufactured in-house, Figure 1A) with the following dimensions: 1 mm thick, 10 mm diameter outer ring, 7 mm diameter inner ring, 18 mm wing to wing. Total weight: 500 mg
8. Craniotomy cover (manufactured manually, Figure 1B) with roughly the following dimensions: ~10 mm × 15 mm for the metal wire looped around the headplate, covered by either tape or hard plastic of ~70 mm^2^
9. Glass window construct
  - Position custom steel rings for the cranial window upside down.
  - Using a paper point, apply a small amount of optical adhesive (NOA 68, Norland Products, catalog number: 25882E) to the bottom rim of the steel ring.
  - Place a cover glass (CS-3R-0 Ø 3 mm #0 thickness, Warner Instruments, catalog number: 64-0726) on the bottom side of the steel ring (Figure 1C).
  - Apply a small pressure with the forceps and make sure the optical adhesive completely connects the steel ring and the cover glass, i.e., without air gaps.
  - Cure the optical adhesive under a UV lamp according to manufacturer’s specifications (3 min under 100 W).
10. Ground pin construct
  - Cut the silver wire (Advent, catalog number: AG549311) to ~3 cm and strip off the Teflon coating.
  - Remove the plastic from the PCB pin and trim the end.
  - For extra mechanical stability, the silver wire could be looped with both ends of the wire soldered together onto the pin.
  - Chlorinate the tip of the wire with bleach for a few minutes and then rinse the bleach off with demi water (Figure 1D).
    **Figure 1.**
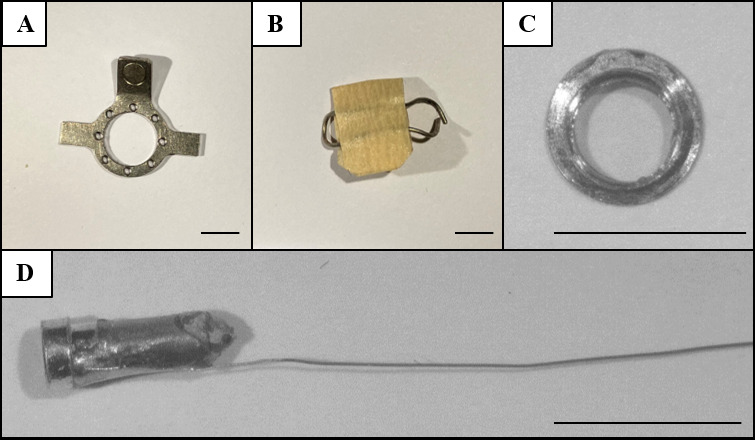
In-house made materials. Shown are the (A) headplate, (B) craniotomy cover, (C) glass window construct, and (D) ground wire construct. Scale bars correspond to 5 mm.

## Equipment

1. Scalpel holder with #10 scalpel blade
2. Coarse forceps
3. Fine forceps (e.g., Fine Science Tools, catalog number: 11254-20)
4. Fine scissors (e.g., Fine Science Tools, catalog number: 15024-10)
5. Micro curette (Fine Science Tools, catalog number: 10082-15)
6. Composite instrument (e.g., Hu-Friedy, catalog number: CI0135)
7. Coverslip forceps (Fine Science Tools, catalog number: 11251-33)
8. Headplate holder (manufactured in-house)
9. Heat pad (e.g., FHC, Bowdoinham, catalog number: 40-90-8C)
10. Dental drill (e.g., Foredom Micromotor, H.MH-170)
11. LED composite curer (e.g., GDT, catalog number: 077340)

## Procedure

- **Headplate installation**
  1. Anaesthetize the mouse with isoflurane (~0.8 L/min, 5%).
  2. Once unconscious, remove mouse from incubation chamber and put under face mask and on a heating pad.
  3. Switch to ~0.8 L/min, 2% isoflurane, and adjust as necessary throughout the surgery.
  4. Apply eye ointment.
  5. Inject carprofen (5 mg/kg) and buprenorphine (0.05 mg/kg) subcutaneously.
  6. When placing a cranial window: Inject 15% D-mannitol (2,000 mg/kg) intraperitoneally.
  7. Straighten the head by supporting the neck with a roll of paper tissue or by using ear bars.
  8. Wet the fur on the scalp using lidocaine spray and shave the scalp with a scalpel.
*Note: Be careful to keep the lidocaine from flowing to the nose of the animal, which will block its airway.*
  9. Use 70% ethanol to sterilize skin, spray lidocaine on clean scalpel, and cut open the skin from 1–2 mm caudal to the occipital ridge to the middle of two eyes.
  10. Spray additional lidocaine on the incision site and exposed periosteum.
  11. Pull skin laterally to expose the skull; cut the periosteum between the skin and the skull following the perimeter of the incision (see Figure 2A).
  12. Locate the anatomical landmarks Bregma and Lambda on the exposed skull.
  13. Make a small incision caudally along the midline to separate the thin membranous muscles at the dorsal neck region. Push away the muscles laterally to expose the caudal edge of the skull (occipital ridge) and the attached muscles (splenius capitis and semispinalis capitis muscles).
  14. Detach the muscles from the occipital bone by cutting with the fine scissors or scrapping with forceps (see Figure 2B). Avoid damaging the muscle by keeping the incisions as close to the occipital bone as possible.
  15. Scratch the bones with a curette or the tip of a scalpel to remove periosteum and residual attached muscle. The exposed area should cover the dorsal surface of the skull from the rostral side to Bregma and the caudal edge of the skull and extend laterally.
  16. Clean the surface with 70% ethanol. Let the bone air dry briefly.
  17. Insert four paper points between the occipital bone and skin and underneath the muscle on the interparietal bone. This makes the area accessible for etching and prevents damage to skin and muscles (see Figure 2C).
  18. Apply etching gel on the skull and let it sit for 15–30 s until the bone surface becomes lighter and turns matte. Wipe away the etching gel with a cotton swab wet with saline until no etching gel is left on the surface of the skull.
  19. Apply bonding primer (OptiBond prime) to the skull using a micro applicator and let it air dry.
*Note: Make sure no excess amount of primer remains on the surface, i.e., it should not look too watery but can be a bit shiny. However, a desiccated over-dried surface will also hamper proper bonding in the next steps.*
  20. Apply bonding adhesive (OptiBond adhesive) to the entire exposed skull with a micro applicator or a paper point in a *scratching* motion while applying some pressure.
*Note: The scratching action ensures that the adhesive gets into small pores of the bone and prevents these pores from closing up.*
  21. Cure adhesive with UV light (see General note 1).
  22. Apply bonding adhesive (OptiBond adhesive) to the headplate using the same micro applicator.
  23. Position the headplate on the skull, so that the opening is slightly caudal to Lambda. Make sure the headplate is as close to the skull as possible. Too much adhesive between the headplate and the skull may prevent short working distance objectives from reaching the brain.
*Note: Positioning depends on the dimensions of the headplate and the purpose of experiments. A well-centered (lateral) headplate eases head fixation for chronic measurements and helps with standardization of position.*
  24. Cure the headplate to the skull with UV light. Ensure that the headplate does not change position while curing.
  25. Secure the headplate with dental composite (see General notes 2 and 3). Make sure all gaps between the headplate and the skull are filled (see Figure 2D).
    - Ensure the dental composite has solid contact with the back part of the skull (occipital bone) in addition to the top parts of the skull (parietal and interparietal bones). This gives extra strength to the skull, prevents accidental breaking during head fixation, and helps reduce relative motion between the skull and the brain during recordings.
    - Fill up the rostral part of the skull with dental composite, with a bit *grabbing onto* the headplate. Be careful not to overdo it as this will increase the imaging distance from the objective.
  26. Close up the opening by attaching any loose skin to the caudal side of the headplate with the superglue.
    **Pause point:** If desired, the craniotomy steps (sections B–D) can be done in a second surgery at a later date. In that case, apply and cure a thin layer of flowable dental composite on the exposed skull to protect the bone and then proceed to section E for recovery.
    **Figure 2.**
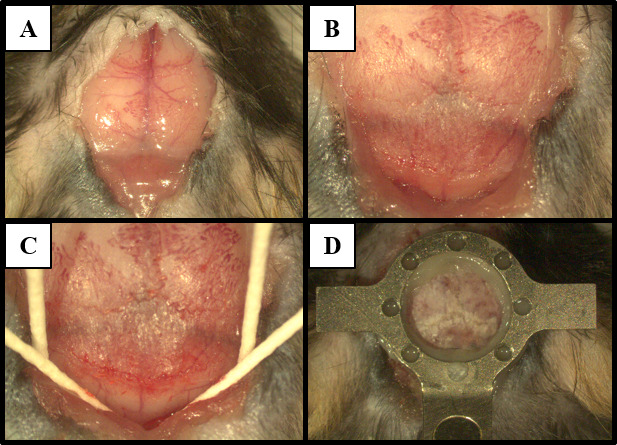
Headplate installation. Images show procedure steps corresponding to (A) exposure of the skull, (B) detachment of the muscles, and (C) preparation of the skull surface for (D) headplate placement roughly over lambda.
- **Craniotomy**
  1. Use landmarks or atlas coordinates to locate the skull region underneath which the IC is expected to be.
  2. Using a fine drill, sequentially thin away the skull (see General notes 4 and 5). Remove any bone debris.
  3. Once the vascularized middle layer has been excavated, apply a bit of saline to the bone. At this point, the bone should be transparent and mildly flexible once pressured with forceps or the drill tip.
  4. At this point, the IC is roughly visible. Use the cerebellum and transverse sinus as landmarks. The cerebellum tends to have small blood vessels running in the sagittal direction.
  5. For cranial window installation (e.g., steel ring constructs): check the position of the IC and extend the size of the craniotomy around it to accommodate the window. This is best done by first marking the intended extent of the craniotomy by drilling with the aid of a glass coverslip glued to a paper point. Because of the skull thickness, add an extra margin of approximately 0.3 mm at the skull surface to ensure the bottom of the craniotomy will fit the cranial window.
  6. Continue to thin the bone above the IC until it becomes membranous. The created funnel should extend rostro-laterally towards the sinus and a bit caudo-medially towards the cerebellum.
  7. Once the intended area is thinned, remove the debris and rinse the surface with saline. Make sure that at this point any bleeding has been stopped to ensure a blood-free brain surface when the bone is removed.
  8. Apply saline on the bone. Using a pair of fine forceps, very carefully chip away the bone above the brain starting from the cerebellum side (see General note 6).
  9. The IC should be rather white and have a shiny surface, compared to the more gray/dark tone of the cerebellum. The dura mater should be intact at this point, forming a continuous surface on top of the IC and the cerebellum.
*Note: Continue to either section C or D; the protocol is not compatible to combine both.*
- **Variant 1: cranial window for in vivo two-photon calcium imaging**
  1. For pre-prepared cranial window constructs (e.g., steel ring constructs), the craniotomy should already fit the size of the cranial window. If necessary, expand the craniotomy and avoid debris and bone shards on the brain surface.
  2. Fit the cranial window within the craniotomy (see General note 7).
  3. Apply pressure and hold the window in place. Apply a tiny drop of superglue with the tip of a paper point to 2–3 contact points between the rim of the window and the bone.
  4. Proceed to secure the cranial window with dental adhesive or cement (see Figure 3). Applying pressure to the cranial window is important to prevent growth of connective tissue between the glass window and the brain. Rule of thumb: if you think you have pressed too much, you are good. Alternatively, the whole window can be fastened with superglue for a removable window.
    **Figure 3.**
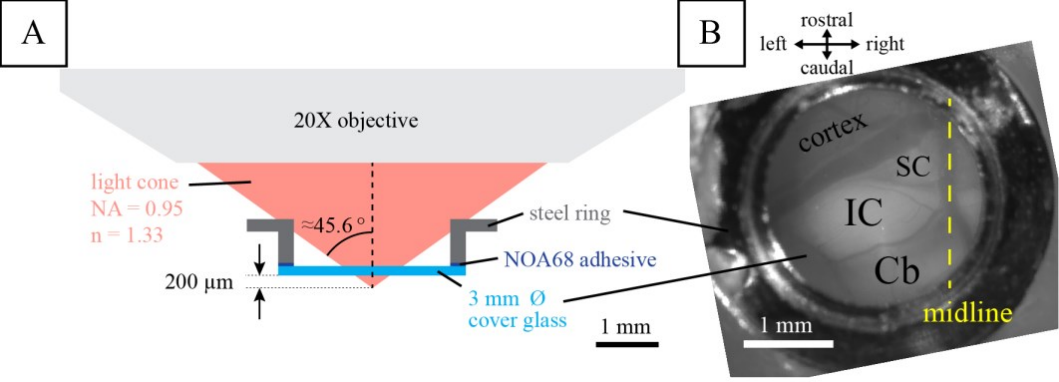
Cranial window for in vivo two-photon calcium imaging. Illustrated is (A) the two-photon calcium imaging setup previously used and (B) the final result of the installed cranial window over the left inferior colliculus (IC). SC, superior colliculus; Cb, cerebellum. Figure reproduced from Wong and Borst (2019) under a CC-BY license.
  5. When applicable: Close the lid of the headplate once the glue has dried.
- **Variant 2: craniotomy for in vivo electrophysiological recordings**
  1. Apply bonding primer to the edges of the craniotomy using a paper point and let it briefly air dry.
  2. Apply bonding adhesive to the craniotomy funnel using a paper point and cure it with UV.
  3. Create a well of dental composite around the craniotomy and cure with UV light.
  4. Secure the ground pin outside or on the edge of the headplate with Charisma. Gently slide the wire in-between the brain and the skull (see General note 9).
  5. Fill the well with the silicone gel. Remove bubbles and debris from the gel using fine forceps.
  6. Allow the silicone gel to set for 30 min.
  7. Secure the ground construct with dental composite, leaving the pin opening exposed. Form a sheet of dental composite that covers the ground construct and the rest of the exposed skull (see General note 2, Figure 4A).
  8. Make a cover by looping wire around the wings of the headplate (see Figure 4B).
    **Figure 4.**
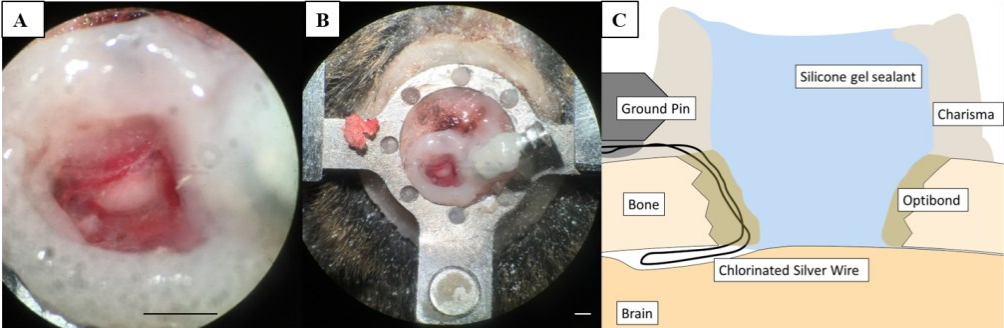
Craniotomy for in vivo electrophysiology recordings. (A, B) Craniotomy over the inferior colliculus (IC) with silicone gel sealant. (C) Schematic overview of a vertical section through the preparation illustrating the Charisma barrier necessary to hold the silicone gel sealant and the position of the ground pin. The secured chlorinated silver wire is placed at the edge of the craniotomy and slipped in between the skull and the brain. Scale bars correspond to 1 mm.
- **Recovery from surgery**
  1. When using D-mannitol, inject saline intraperitoneally at a volume equal to previously injected D-mannitol.
  2. Stop isoflurane and allow the mouse to wake up.
  3. Wait for the first sign of spontaneous movements and transfer the animal to its cage under a heat lamp or on a heating pad.
  4. For electrophysiological recordings, allow animals to recover for 3–4 days before performing subsequent awake experiments (Figure 5). For in vivo two-photon calcium imaging, imaging can start after 3–4 days of recovery. However, for stable, repeated long-term recordings, it is advisable to wait for two weeks after the surgery before imaging to allow the brain tissue to adapt and stabilize mechanically.
    **Figure 5.**
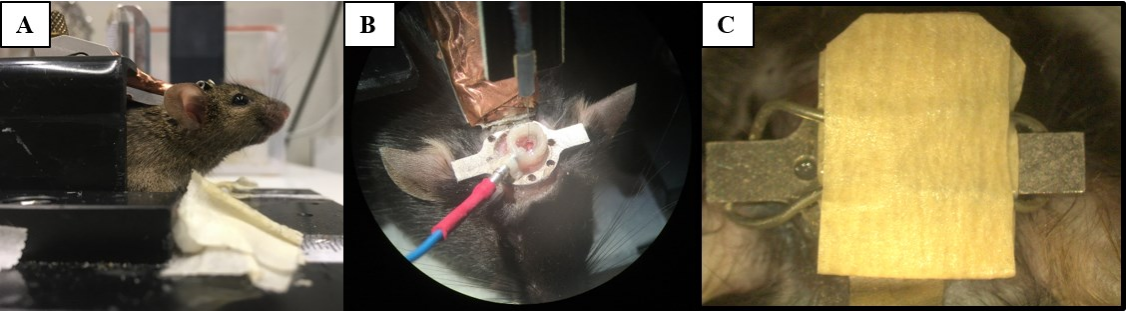
Head fixation and awake electrophysiological recordings. During electrophysiological recordings, mice are head fixed (A) before the recording electrode can be lowered into the craniotomy and the ground pin connected (B). To protect the gel sealant, the craniotomy is covered (C).

## Validation of protocol

The protocol as described was used in previous works; see van den Berg et al. (2023) for its implementation in in vivo electrophysiology experiments and Wong and Borst (2019) for in vivo imaging application.

In van den Berg et al. (2023), we performed electrophysiological recordings using a high-density acute silicon probe single-unit recordings (see Figure 1B and sections “Surgery” and “Electrophysiological Recordings” from the materials and methods section in the cited article). The probe was mounted on a 3-axis motorized micromanipulator (Luigs-Neumann Mini-25 controlled by a SM-8 controller) and inserted perpendicular to the brain surface. The probe was connected to a head stage (RHD 64-channel headstage, part C3315, Intan Technologies) and acquisition board (RHD USB-interface board, part C3100, Intan Technologies, Los Angeles, CA). The electrophysiological signal was sampled at 30 kHz using the supplied recording GUI (RHD data acquisition GUI, Intan Technologies). At the end of the experiment, the probe was slowly retracted from the craniotomy and cleaned by placing the tip in protease-containing detergent (e.g., 1% Tergazyme) for 1 h followed by 1 h in ddH_2_O. This protocol was used for the awake recording data set in van den Berg et al. (2023), which amounted to 99 well-isolated single units recorded from four animals across recording sessions spanning between 4 and 13 days.

**Figure 6.**
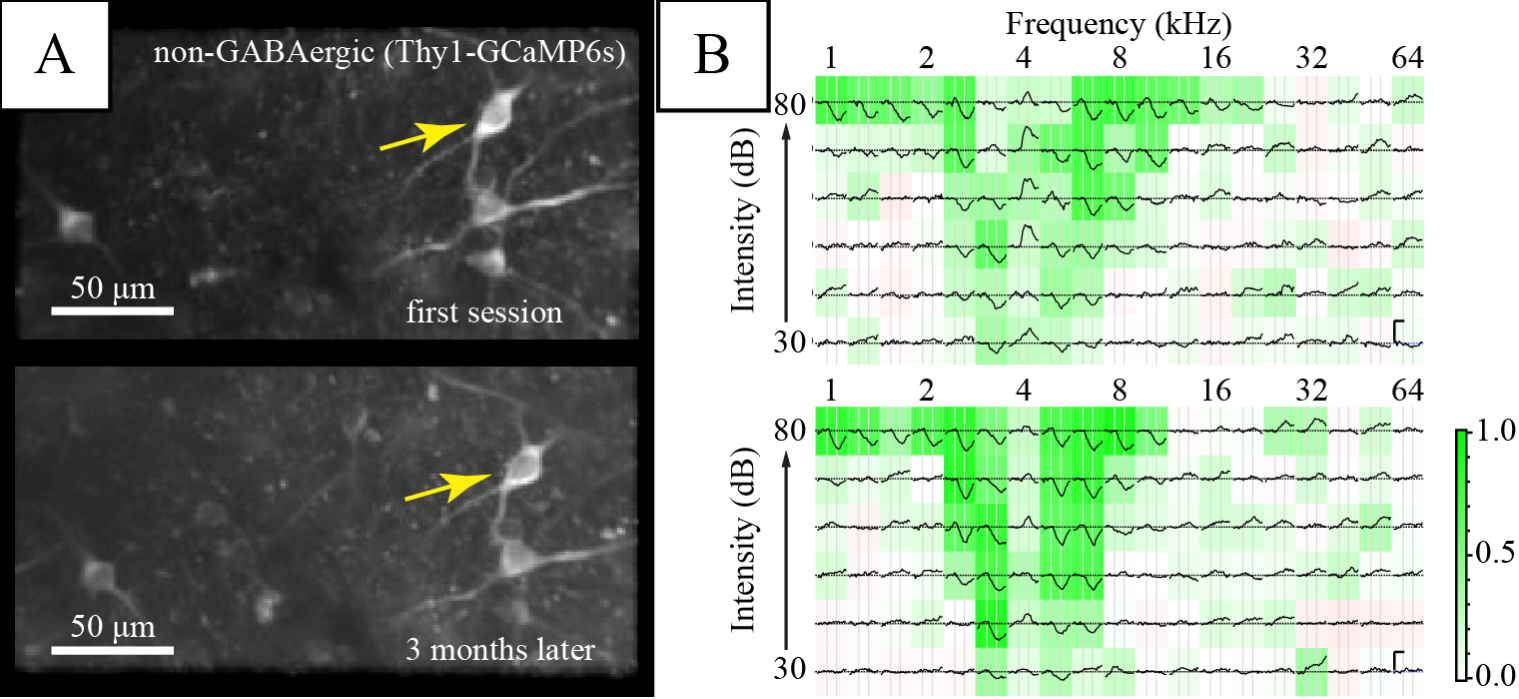
Long-term imaging of the same inferior colliculus (IC) neurons. (A) Average fluorescence images of a region of interest in the dorsal IC. (B) Frequency response areas of the neuron marked by the yellow arrow in the corresponding session. Each subplot shows the average ΔF/F_b_ to a stimulus of the specified frequency and intensity. Background color shows average Pearson’s correlation among repetitions, indicating consistency of response (see Geis et al., 2011). Vertical scale bars in the right panel indicate 1 F_b_. Horizontal scale bar is 1 s.

In Wong and Borst (2019), we performed in vivo two-photon imaging using a custom-built two-photon microscope with a 20× water-immersion objective (LUMPlanFI/IR, 20×, NA: 0.95; Olympus Corporation) (see Figure 1, “surgery” and “Two-photon imaging” from the materials and methods section in the cited article). Excitation light (920 nm) was provided by a MaiTai Ti:Sapphire laser (Spectra Physics Lasers, Mountain View). Images (256 × 128 pixels) were collected at 9 Hz (2 ms/pixel; 1–2 mm/pixel). In addition, this protocol has been used in our lab to measure neuronal activity in the IC for an extended period of time, as shown by pilot data in Figure 6 performed in a GCaMP6s-expressing mouse line (GP4.3; Chen et al., 2013).

## General notes and troubleshooting

**General notes**

1. Illumination from the surgical light may also cure the adhesive and dental composite (Charisma). Turn down the light intensity or block it with orange (UV) filters if it tends to harden too quickly while handling.
2. The dental composite used comes in two viscosities; use standard (paste-like) version for larger surfaces and flowable, low-viscosity version to fill up any small gaps left. Cure with UV light in between layers.
3. Clean tools with 70% ethanol before handling dental composite to prevent it from sticking too strongly to the tools. Preferably, use smooth, unscratched parts of the tools.
4. The bone above the IC (interparietal bone) consists of three layers: the middle layer is more vascularized, while superficial and deep layers are relatively vessel-free.
5. Small bleedings from the bone may be stopped by briefly drilling towards the source; otherwise, applying slight pressure helps to stop bleeding. Stop larger bleedings by applying a small piece of pre-wet Spongostan for a brief period (one to a few minutes) and then remove it slowly and gently with plenty of saline.
6. When removing the bone, working in solution helps prevent damage to the dura mater. For larger pieces that adhere strongly to dura, use two pairs of fine forceps: pick up a piece of bone on one side with the bent forceps and very slowly lift it up bit by bit like opening a rooftop trapdoor. The other fine forceps can be used to gently detach the bone from the connective tissue in a way similar to blunt dissection techniques.
7. For prepared cranial windows constructs (e.g., double glass, steel rings), make sure the cranial window fits into the opening before actually opening up the bone. This will avoid having to drill or chip away more bone at the edge later on, which may add bone shards on the brain surface and interfere with imaging.
8. A blood-free brain surface is highly beneficial to imaging. Try to prevent the formation of blood clots by promptly rinsing away any blood on IC surface. Try to remove any clotting blood with forceps before putting the glass window in.
9. During electrophysiological recordings, standard measures should be taken to reduce electrical noise such as proper grounding and shielding from electrical interference. For this preparation, the proximity of the ground wire to the recording site reduces impedance in general. Securely cementing the ground wire helps to reduce motion artefacts. The use of Ag/AgCl electrodes reduces offset drifts and makes this protocol also suitable for patch-clamp type recordings.
10. Daily maintenance of the imaging glass window involves rinsing the glass surface with saline and removing it with a Sugi or suction. In case of dirt or dried up fluid that is difficult to remove, a gentle scratch with fine forceps in saline can help dislodge it. A deterioration in the quality of view under the window, i.e., between the glass and brain, is mostly due to a (re-)growth of tissue between the glass and the brain. There is no good, practical way to reverse this process once happened. Thus, it is important to minimize the chance by making sure that no bone shards remain on the brain surface and applying enough pressure while fixing the cranial window. See also problems 2 and 3 under Troubleshooting.

**Troubleshooting**

Problem 1: Headplate detaches from the skull after a few days or upon head fixation.

Possible cause #1: Skull became weak due to immune response.

Solution: Make sure the procedure is performed as cleanly as possible. Sterilize all tools and disinfect skin and fur of the animal around the incision. If desired, dexamethasone can be given to the animal at/after the surgery to reduce immune reaction.

Possible cause #2: Adhesive did not cure properly.

Solution: Make sure the skull surface is dry. Make sure the adhesive and primer have not expired. Make sure the adhesive or primer were not contaminated by other chemicals (e.g., ethanol).

Possible cause #3: Poor contact between the dental composite and the skull.

Solution: To prevent this, make sure that, when placing the headplate, the skull surface is dry and any tissue is removed. A headplate with small holes in the top can help to fill up the space between the headplate and the skull with the low viscosity composite. When securing the headplate with dental composite, pay extra attention to securing the back of the skull.

Problem 2: Significant (re-)growth of soft tissue between brain surface and cranial window, impairing imaging.

Possible cause #1: Not enough pressure between glass window and brain surface.

Solution: Make sure sufficient pressure was applied when securing the window. If necessary, thin the bone where the outer rim of the steel ring will sit to allow a deeper insertion of the window construct.

Possible cause #2: Bone growth between glass window and brain surface.

Solution: See Problem 3.

Problem 3: Growth of bone between brain surface and cranial window.

Possible cause: Small bone shards between cranial window and brain surface began to grow and enlarge.

Solution: Clear the brain surface of any tiny bone shards before installing cranial window.

Problem 4: Detachment of ground pin from the animal.

Possible cause: The animal could have bumped into something in the home cage that made the pin detach. Any liquid or air pockets make the attachment of the dental composite less secure.

Solution: In general, take care to position the ground pin as to not protrude out of the headplate too much/at all. Make sure a sufficient amount of dental composite surrounds the ground pin. To remedy an animal with a detached pin, check the state of the craniotomy and confirm that brain was not damaged by the event. In that case, attach a new pin and electrically connect it to the end of the remaining silver wire using conductive paint such as RS PRO Conductive Lacquer.

Problem 5: Cloudy glass window.

Possible cause: Vapor of superglue will condense on the clean surface of the glass window and make it foggy.

Solution: This is a problem that occurs often. The vapor is very easily removed by gentle scratching with forceps once the window is fixed. The removal can be done (preferably) at the first imaging session.
